# Thioredoxin Dependent Changes in the Redox States of FurA from *Anabaena* sp. PCC 7120

**DOI:** 10.3390/antiox10060913

**Published:** 2021-06-04

**Authors:** Jorge Guío, María Teresa Bes, Mónica Balsera, Laura Calvo-Begueria, Emma Sevilla, María Luisa Peleato, María F. Fillat

**Affiliations:** 1 Departamento de Bioquímica y Biología Molecular y Celular, Facultad de Ciencias, Instituto de Biocomputación y Física de Sistemas Complejos (GBsC-CSIC and BIFI-IQFR Joint Units), Universidad de Zaragoza, 50009 Zaragoza, Spain; jguio@unizar.es (J.G.); tbes@unizar.es (M.T.B.); Lmcalvob@aragon.es (L.C.-B.); esevilla@unizar.es (E.S.); mpeleato@unizar.es (M.L.P.); 2 Instituto de Recursos Naturales y Agrobiología, Consejo Superior de Investigaciones Científicas, 37008 Salamanca, Spain; monica.balsera@csic.es

**Keywords:** cyanobacteria, ferric uptake regulator A, thioredoxin

## Abstract

FurA is a multifunctional regulator in cyanobacteria that contains five cysteines, four of them arranged into two CXXC motifs. Lack of a structural zinc ion enables FurA to develop disulfide reductase activity. In vivo, FurA displays several redox isoforms, and the oxidation state of its cysteines determines its activity as regulator and its ability to bind different metabolites. Because of the relationship between FurA and the control of genes involved in oxidative stress defense and photosynthetic metabolism, we sought to investigate the role of type *m* thioredoxin TrxA as a potential redox partner mediating dithiol-disulfide exchange reactions necessary to facilitate the interaction of FurA with its different ligands. Both in vitro cross-linking assays and in vivo two-hybrid studies confirmed the interaction between FurA and TrxA. Light to dark transitions resulted in reversible oxidation of a fraction of the regulator present in *Anabaena* sp. PCC7120. Reconstitution of an electron transport chain using *E. coli* NADPH-thioredoxin-reductase followed by alkylation of FurA reduced cysteines evidenced the ability of TrxA to reduce FurA. Furthermore, the use of site-directed mutants allowed us to propose a plausible mechanism for FurA reduction. These results point to TrxA as one of the redox partners that modulates FurA performance.

## 1. Introduction

Fur (ferric uptake regulator) is a master transcriptional regulator in prokaryotes, which usually modulates a broad set of genes not only related to the control of iron homeostasis. In heterotrophic bacteria, Fur has been found to be involved in the regulation of the oxidative stress response, biofilm formation, control of host–pathogen interaction, and modulation of central metabolism, among other processes [1]. The initial working model proposed that Fur acted as a classical repressor, which required Fe^2+^ to bind target DNA-sequences (iron-boxes) [2]. However, further work unveiled that Fur is a multifaceted regulator, sensitive to oxidants, which can activate transcription and that, in some cases, also binds DNA in the absence of iron [3,4,5,6].

The available structures of Fur proteins from different organisms unveil that, in addition to the regulatory metal-binding site, Fur displays a so-called structural zinc-binding site which, in most cases, is formed by four cysteines arranged in two CXXC motifs [1]. Furthermore, it has been found that, in *E. coli*, Fur binds a [2Fe-2S] cluster via conserved cysteine residues [7]. Fur has also been identified as one of the transcription factors that are associated with thioredoxin in a detailed analysis of the thioredoxin-linked *E. coli* proteome [8], as well as in further studies using *E. coli* engineered strains to optimize trapping and characterization of the in vivo Trx redox interactome [9]. Therefore, Fur activity seems to be tightly related to iron and redox signaling and associated with the regulation of ROS-responsive genes, sometimes in a coordinate manner with other redox-responsive regulators [10,11,12]. This cross-talk between iron homeostasis and redox regulation is especially critical in nitrogen-fixing cyanobacteria, where the photosynthetic electron transport chain and the nitrogenase complex are rich in iron-sulfur clusters, which are quite labile in the presence of oxidants [13]. Furthermore, genetic and physiological studies in the cyanobacterium *Anabaena* sp. PCC7120 unveil a tight relationship between FurA and the photosynthetic metabolism [14]. In much the same way, in addition to controlling a plethora of photosynthesis-related genes, a functional photosynthetic electron transport chain is required for the expression of the FurA ortholog in *Microcystis aeruginosa* PCC7806 [15]. 

Unlike most Fur family proteins, despite containing two CXXC motifs, FurA from *Anabaena* sp. PCC7120 does not contain zinc [16], a fact that allows the occurrence of intra and intermolecular dithiol-disulfide exchanges reactions. Thus, FurA behaves as a redox regulator integrating iron homeostasis with the redox status of its five cysteines, namely C101, C104, C133, C141, and C144 [3,17]. FurA CXXC motifs are located at positions C101XXC104 and C141XXC144. Our previous work showed that recombinant FurA contains two intramolecular disulfide bridges, namely C104-C133 and C141-C144 [17]. In addition, FurA displays the features of a heme-sensing protein through the interaction of C141 as an axial ligand of Fe (III) high-spin heme [18]. Moreover, FurA binds 2-oxoglutarate (2-OG) in vitro, enhancing FurA binding to the *ntcA* promoter [19]. Although in this case an arginine residue has been implicated in the complex formation, cysteines appear to be important in the process since the effective binding of both species, in addition to manganese, requires the presence of a reducing agent. In fact, in the absence of manganese and/or DTT, FurA is unable to interact with 2-OG [19]. Furthermore, the binding of FurA to the co-repressor metal depends on the redox status of C101 residue, which is part of a thiol/disulfide redox switch that determines FurA ability to bind this metal and hence to DNA [17]. Interestingly, FurA shows several redox states in the cyanobacterial cytosol [20] and redox-proteomic studies aimed to identify targets of light/dark modulation in unicellular cyanobacteria found that the FurA orthologs from these cyanobacteria undergone dynamic thiol redox changes [21,22,23]. In spite of all of this evidence, the precise mechanism underlying the reduction of FurA, as well as its functional electron donor (s), remain still unknown. 

Since thioredoxins are essential players in thiol-based redox regulation and are involved, among many other processes, in the regulation of the activity of many redox-sensitive transcription factors [24], we sought to investigate the potential role of thioredoxin type-*m1* (named TrxA) in the redox modulation of FurA. Although photosynthetic organisms contain at least 19 types of thioredoxins, TrxA is the most abundant one among the major types of thioredoxins present in chloroplasts and cyanobacteria and one of the major hubs in their redox networks [25]. Thioredoxins from *E. coli* and those present in the cytosol and mitochondria of higher plants are reduced by a NADPH-dependent thioredoxin reductase (NTR) [26]. Conversely, it is accepted that in photosynthetic organisms the main pathway for the reduction of TrxA involves electron flux from the reduced photosynthetic electron transport chain, which in turn transfers electrons to the ferredoxin:thioredoxin reductase (FTR) system [27]. In addition, some cyanobacteria, such as *Gloeobacter* and *Prochlorococcus*, lack NTR and FTR but contain a thioredoxin reductase flavoenzyme called FFTR, which receives electrons form ferredoxin [28]. Interestingly, in vitro, cyanobacterial TrxA can also accept electrons from NTR from *E. coli* [29,30].

In this work, we report a hitherto unknown functional relationship between the global regulator FurA and TrxA in cyanobacteria. Our results demonstrate that TrxA interacts in vivo with FurA from *Anabaena* sp. PCC7120, as assessed using a bacterial two-hybrid system, and we show that TrxA is able to reduce FurA. It is well established that thioredoxins reduce their target proteins by dithiol exchange of their conserved WCGPC active site motif to a disulfide of the target. Since recombinant FurA contains two disulfide bonds, by using different triple site-directed cysteine mutants that conserve only one of these disulfides, we show that thioredoxin displays a preference for one of them and propose a plausible mechanism for the reduction of this multifunctional transcriptional regulator by thioredoxin. 

## 2. Materials and Methods

### 2.1. Cloning, Expression and Protein Purification

The *furA* wild-type gene (*all1691*) from *Anabaena* sp. PCC7120 was amplified from *Anabaena*′s DNA and ligated into pET28a (Novagen: Merck, Darmstadt, Germany) as previously described [3]. pET28a-*furA* constructs of mutants C101S-C141S-C144S and C101S-C104S-C133S were purchased from Mutagenex (Piscataway, NJ). Recombinant expression and purification of FurA wild-type and its mutants were performed as previously described [31]. 

The *trxA* gene was amplified from *Anabaena*’s DNA with *Pfu* Ultra polymerase using primers alr0052up (5´GGAATTCCATATGTCAGCAGCCGCACAAGTTACAGATTC3′) and alr0052dw (5´CCCAAGCTTTCACAGATGCTTTTCTAG 3′). After denaturation at 95 °C for 5 min, 30 cycles of denaturation for 30 s, annealing at 59 °C, and polymerization at 72 °C for 5 min were carried out. The resulting amplification product was digested with NdeI and HindIII, ligated to pET28a, and introduced into *E. coli* BL21 (DE3). Induction of the recombinant protein was performed at the late exponential phase of growth using 0.5 mM IPTG for 4 h. The recombinant protein was purified from 10 g of biomass resuspended in 50 mL of 50 mM Tris/HCl pH 8.0, 500 mM NaCl, and 2 mM PMSF. Cells were disrupted by sonication (20 kHz, 80 W) on ice for 4.5 min in periods of 45 s. Lysed cells were centrifuged at 4 °C at 48,000× *g* for 20 min. The resulting supernatant was centrifuged two more times in identical conditions. The crude extract was filtered through 0.45 μM Millipore filters and loaded onto a 10 mL affinity column of Chelating Sepharose Fast flow matrix (GE Healthcare) previously loaded with 0.25 M ZnSO_4_ and equilibrated with 50 mM Tris/HCl pH 8.0, 500 mM NaCl (buffer A). Afterwards, the column was first washed with the same buffer supplemented with 0.5 M (NH_4_)_2_SO_4_ and then with the same buffer supplemented with 35 mM glycine until no protein was detected (Abs _280 nm_ < 0.1). Finally, the purified protein was eluted with a gradient of imidazole 0 to 1 M in buffer A. Samples were collected in aliquots of 1 mL, verified by SDS-PAGE, and stored at −20 °C until use. 

NTR from *E. coli* was prepared as described previously [30]. Briefly, NTR with a His-tag was produced in Rosetta (DE3) *E. coli* cells and purified from the soluble extract by Ni^2+^ affinity chromatography. The tag was removed by a His-tagged TEV protease, incubated with an excess of FAD, and further purified by gel filtration using a Sephacryl S-300 HR column (Cytiva) equilibrated in 20 mM Tris-HCl pH 7.6, 150 mM NaCl buffer. 

The flavodoxin gene from *Anabaena* sp. PCC 7120 was amplified by PCR, cloned in pTrc99b, overexpressed, and the recombinant protein purified as described in [32]. Recombinant ferredoxin-NADP^+^-reductase (FNR) was a generous gift of Dr. M. Martínez-Júlvez. 

### 2.2. FurA Cross-Linking Assays

Equimolar concentrations of FurA and TrxA (20 μM), as well as ferredoxin-NADP^+^-reductase and flavodoxin (positive control) [33], and negative controls composed of FurA plus FNR and TrxA plus FNR, were incubated for 10 min in 50 mM Tris-HCl pH 8.0 prior to the addition of 2 mM 1-Ethyl-3-(3-dimethylaminopropyl) carbodiimide (EDC) and 2 mM N-hydroxysuccinimide (NHS). The crosslinking reaction was again incubated for 20 min and then the crosslinked solution was mixed with Laemmli buffer, boiled, and resolved on 15% gels by SDS-PAGE. The gel was stained with Coomassie and destained to visualize the proteins.

### 2.3. Reconstitution of the NTR/TrxA/FurA Redox Cascade and Determination of Protein Redox States In Vitro

FurA and its mutants (1 μM) were incubated with 0.5 μM TrxA, 0.5 mM EDTA, 0.2 mM NADPH, and 5 nM NRT in a final volume of 800 μL of 50 mM Tris-HCl pH 8.0. Proteins were also incubated with 0.2 mM NADPH and 5 nM NRT to determine whether NADPH-reduced NTR affected the redox status of FurA or its mutants and with 1 mM 1,4-dithiothreitol (DTT) as a positive control for protein reduction. After incubation for 15 min at 25 °C, samples were divided into two aliquots. Proteins were precipitated with 10% (*w*/*v*) trichloroacetic acid (TCA) for 15 min on ice and subsequentially centrifugated at 8000 g for 10 min at 4 °C. Pellets were washed with cold acetone and precipitates were resuspended in 20 μL of 50 mM Tris-HCl pH 8.0, 1% (*w*/*v*) SDS with or without 20 mM 4-acetamido-4´-maleimidylstilbene-2,2´-disulfonic acid, disodium salt (AMS, Invitrogen). After 2 h of treatment, samples were subjected to 15% non-reducing SDS-PAGE. Gels were stained with Coomassie and destainedto visualize the proteins.

### 2.4. Determination of FurA Redox States In Vivo 

*Anabaena* sp. PCC7120 cells at mid-exponential phase (optical density at 750 nm = 0.5–0.6) were grown photoautotrophically at 25 °C in BG-11 medium using 250 mL Erlenmeyer flaks containing 100 mL of culture medium. These cultures were maintained in a New Brunswick™ Innova^®^ 43 Shaker under continuous illumination (30 μmol photons m^−2^ s^−1^ of white light) at 25 °C and gentle shaking at 100 rpm. For the study of light-dark modulation of FurA redox status, cultures were wrapped in aluminum foil for 15 min and then cells were re-exposed to light for 3 h with or without 2 mM DTT. For every condition, 600 μL of cells were harvested and incubated with TCA to a final concentration of 1% (*v*/*v*) for 15 min on ice to avoid artificial thiol-disulfide modifications [34]. After centrifugation at 8000 g for 10 min at 4 °C, pellets were washed with cold acetone and divided into two aliquots. One of them was resuspended in 20 μL of 50 mM Tris/HCl pH 8.0, 1% (*w*/*v*) SDS and the other one in the same buffer containing 20 mM AMS (Molecular Probes). After 2 h of treatment, samples were subjected to 15% non-reducing SDS-PAGE and revealed by Western blotting. For Western Blot analyses, SDS-PAGE gels were transferred onto PVDF filters and then membranes were blocked overnight with TBS-T buffer (50 mM Tris-HCl pH 7.5, 150 mM NaCl and 0.05% Tween-20) containing 5% non-fat dry milk powder. Membranes were washed three times with TBS-T buffer and incubated with rabbit polyclonal antibodies raised against FurA in TBS-T for 1 h. After that, membranes were washed again three times with TBS-T and incubated with anti-rabbit IgG antibodies labelled with peroxidase (Applied Biological Materials Inc.) in TBS-T for 1 h. Finally, proteins were revealed with the Pierce ECL Western Blotting Substrate (Thermo Scientific, Waltham, MA, USA) in an Amersham™ Imager 600 imaging system (GE HealthCare) and band density analyzed by the QuantityOne software (Bio-Rad).

### 2.5. Analysis of Protein–Protein Interaction Using the Bacterial Two-Hybrid System

In vivo protein-protein interaction was assessed using a bacterial two hybrid system (BACTH) based on adenylate cyclase [35]. BACTH system kit was purchased from Euromedex and used as recommended. The *furA* and *trxA* genes of *Anabaena* sp. PCC7120 were amplified from *Anabaena*´s DNA with Expand™ Long Template PCR System (Sigma-Aldrich) using primers DH-FurA-fw 5′CGCGGATCCCATGACTGTCTACACAAATACTTC3′ and DH-FurA-rv 5′CGGGGTACCCGAAGTGGCATGAGCGCACGTTGGC3′ in the case of *furA* and DH-TrxA-fw 5′CGCGGATCCCATGTCAGCAGCCGCACAAGT3′ and DH-TrxA-rv 5′CGGGGTACCCGCAGATGCTTTTCTAGGGTTTGA3′ in the case of *trxA*. The resulting amplification products were cloned into the KpnI and BamHI sites of pUT18, pUT18C, pKT25, and pKNT25 vectors, using *E. coli* XL1-Blue cells for cloning procedures, obtaining fusion proteins with the T18 or T25 domains of adenylate cyclase on their N- or C-terminal ends. Then, competent *E. coli* BTH101 cells were prepared and co-transformed by the heat-shock method with the eight possible combinations of plasmids, thus obtaining eight different transformants: 1: pKNT25-*trxA* + pUT18-*furA*; 2: pKNT25-*trxA* + pUT18C-*furA*; 3: pKT25-*trxA* + pUT18-*furA*; 4: pKT25-*trxA* + pUT18C-*furA*; 5 pUT18-*trxA* + pKNT25-*furA*; 6: pUT18-*trxA* + pKT25-*furA*; 7 pUT18C-*trxA* + pKNT25-*furA*; and 8: pUT18C-*trxA* + pKT25-*furA*. *E. coli* BTH101 co-transformed with empty pUT18C and pKT25 plasmids served as negative control (C-), whereas *E. coli* BTH101 co-transformed with pKT25-zip and pUT18C-zip served as positive control (C+) for complementation. pKT25-zip and pUT18C-zip contain the leucine zipper of GCN4 fused in frame to the T25 and T18 fragments, respectively, and produce the T25-zip and T18-zip fusion proteins, which yield a strong interaction as a result of dimerization of the leucine zipper motifs appended to the T25 and T18 fragments [35].

An overnight culture of each strain was diluted to a final turbidity of 0.05 in LB medium and incubated at 37 °C under strong aeration. When cultures reached a turbidity of 0.1, protein expression was induced with 0.5 mM of IPTG and cultures were grown for 24 h at 30 °C. Then aliquots were taken and β-galactosidase activity was measured as described by Miller [36]. All assays were performed in triplicate.

## 3. Results

### 3.1. FurA Interacts with TrxA 

#### 3.1.1. Cross-Linking of FurA with TrxA 

**Figure 1:**
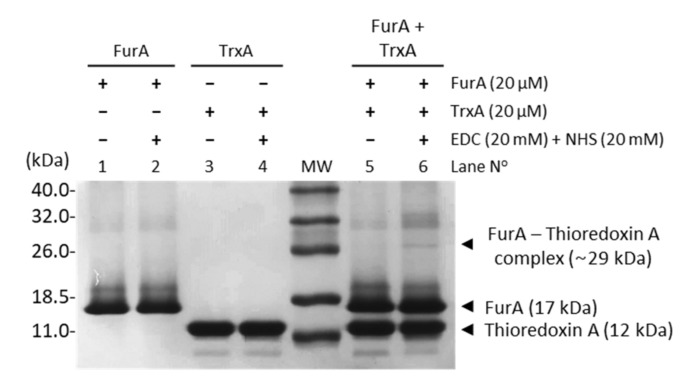
Cross-linking of FurA with TrxA. Equimolar concentrations (20 μM) of proteins pre-incubated with 20 mM EDC and 20 mM NHS were boiled with β-mercaptoethanol and analyzed by SDS-PAGE (15% gel). Samples without the cross-linker agent were used as control.

In order to assess the molecular interaction between FurA and TrxA, cross-linking experiments were carried out in the presence of EDC and NHS. Figure 1 unveils that a small fraction of FurA molecules was successfully cross-linked to TrxA since, upon EDC and NHS treatment, a novel band with molecular mass around 29 kDa, which corresponds to a (1:1) FurA-TrxA complex, can be observed. This result is a representative image of three replicas of the experiment with different pools of proteins from several purifications. In all cases, only a discrete fraction of FurA and TrxA was trapped as a complex, resulting in a faint band compared to the abundant complex detected in a positive control using ferredoxin-NADP+-reductase (FNR) and flavodoxin (Figure S1). Specificity of the cross-linking reaction was confirmed by the absence of cross-linking of FurA with FNR and TrxA with FNR (Figure S1). 

#### 3.1.2. In Vivo Analysis of FurA–TrxA Interaction 

**Figure 2:**
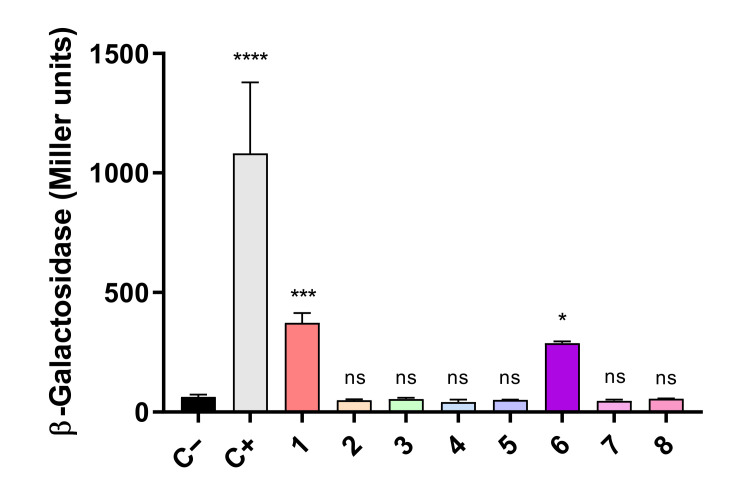
Bacterial two-hybrid assays between FurA and Thioredoxin A. In vivo interaction between FurA and TrxA was quantified by measuring β-galactosidase activities in *E. coli* BTH101 cells harboring a pair of compatible plasmids with corresponding genes fused with the T25 or T18 adenylate cyclase domains: 1: pKNT25-*trxA* + pUT18-*furA*; 2: pKNT25-*trxA* + pUT18C-*furA*; 3: pKT25-*trxA* + pUT18-*furA*; 4: pKT25-*trxA* + pUT18C-*furA*; 5 pUT18-*trxA* + pKNT25-*furA*; 6: pUT18-*trxA* + pKT25-*furA*; 7 pUT18C-*trxA* + pKNT25-*furA*; and 8: pUT18C-*trxA* + pKT25-*furA.* Empty pKT25 and pUT18C served as negative controls (C−), whereas pKT25-zip and pUT18C-zip served as positive controls (C+). The β-galactosidase activity represents the averages ± standard deviation of three biological replicates. All β-galactosidase activity values were subjected to a one-way analysis of variance (ANOVA) test with respect to the negative control (C−) to determine if values were significant (^ns^ not significant, * *p* < 0.05, *** *p* < 0.001, **** *p* < 0.0001).

To verify the interaction between FurA and TrxA in vivo, the adenylate cyclase-dependent bacterial two-hybrid system was used. This system is based on the synthesis of cyclic AMP in double transformants containing the T25 and T18 fragments from the catalytic domain of the *Bordetella pertussis* adenylate cyclase, which are fused to the proteins whose interaction is being analyzed. When this interaction occurs, both fragments of adenylate cyclase are close enough to restore its activity; cyclic AMP is synthesized and binds to the catabolite gene activator protein, which in turn triggers transcription of *lacZ* [35]. Thus, *E. coli* BTH101 cells were co-transformed with pairs of recombinant plasmids expressing the T25 and T18 domains fused to FurA and TrxA in the eight possible combinations, and the efficiencies of functional complementation between the different hybrids were determined by β-galactosidase assays, as described in Materials and Methods. As shown in Figure 2, we observed that two of these combinations yielded transformants with significant β-galactosidase activity, evidencing that FurA associates in vivo with TrxA. As it can be seen in Figure 2, the β-galactosidase activity of these transformants is remarkably lower than the activity of the positive control, suggesting again a weak interaction between FurA and TrxA, as seen in cross-linking experiments.

### 3.2. Thioredoxin A Is Able to Reduce FurA in Anabaena sp. PCC7120

**Figure 3:**
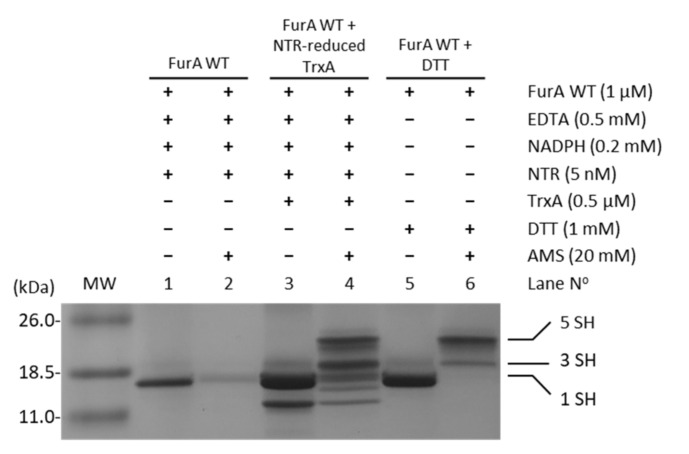
Reduction of FurA by TrxA. FurA WT (1 µM) was incubated with 0.5 µM TrxA in the presence of 0.2 mM NADPH, 0.5 mM EDTA, and 5 nM NTR to assess its reduction by NTR-reduced TrxA. FurA was also incubated with 0.2 mM NADPH and 5 nM NRT to determine whether NADPH-reduced NTR affected its redox status with 1 mM DTT as a positive control for protein reduction. Proteins were then precipitated with 10% (*w*/*v*) TCA, incubated with 20 mM AMS, loaded on non-reducing 15% SDS–polyacrylamide gels, and stained with Coomassie.

In order to assess whether TrxA was able to reduce cysteine residues in FurA, and considering that NTR from *E. coli* was able to reduce *Anabaena* TrxA [29], the electron transport chain NADPH→NTR→TrxA→FurA was reconstituted in vitro. The status of FurA cysteines in the presence of the different components of this system was evaluated using the alkylating agent AMS, which allows the reduced and oxidized forms of FurA to be distinguished. Figure 3 shows that in the absence of TrxA, the reduced/oxidized ratio of thiol/disulfide groups in FurA is not affected (lanes 1 and 2). However, when the complete system is present, TrxA reduced with NADPH/NTR is able to reduce disulfide bridges present in FurA as it can be detected after alkylation with AMS (lanes 3 and 4). In lane 4, the oxidized and reduced forms of TrxA can be identified (two lower bands), as well as several bands corresponding to FurA after reduction with the TrxA system, indicating that TrxA is able to reduce both disulfide bridges in a major fraction of the regulator resulting in five free thiols (upper band). Another fraction of FurA exhibits one reduced disulfide with three free thiols (indicated in Figure 3 as 3 SH) and the rest of the protein remains oxidized showing only one free thiol (indicated in Figure 3 as 1 SH).

### 3.3. Thioredoxin A Preferentially Reduces the Disulfide Bridge between C_141_ and C_144_ in Recombinant Wild-Type FurA

**Figure 4:**
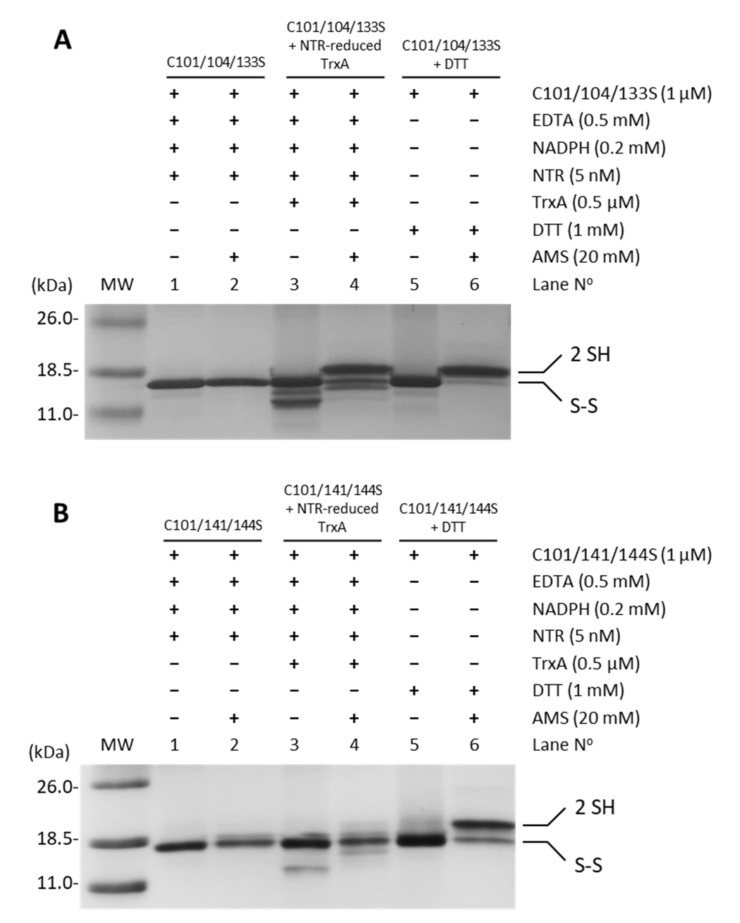
Reduction of disulfide bridges C141-C144 and C104-C133 of FurA by TrxA. One micromolar of FurA triple cysteine mutants C101/104/133S (**A**) and C101/104/133S (**B**) were incubated with 0.5 µM TrxA in the presence of 0.2 mM NADPH, 0.5 mM EDTA, and 5 nM NTR to assess their reduction by NTR-reduced TrxA. FurA triple cysteine mutants were also incubated with 0.2 mM NADPH and 5 nM NRT to determine whether NADPH-reduced NTR affected their redox status and with 1 mM DTT as a positive control for protein reduction. Proteins were then precipitated with 10% (*w*/*v*) TCA, incubated with 20 mM AMS, loaded on non-reducing 15% SDS–polyacrylamide gels and stained with Coomassie.

Oxidized recombinant FurA usually shows two intramolecular disulfide bridges formed by C104-C133 and C141-C144 [17]. In order to identify the TrxA disulfide target, the potential reduction by the NADPH-NTR-TrxA pathway of triple site-directed mutants of FurA keeping one of these disulfides was evaluated. Figure 4 shows that while TrxA efficiently reduces the C141-C144 disulfide bridge (Figure 4A), thiol-disulfide exchange with the pair C104-C133 is not performed (Figure 4B). This result points to the C141-C144 disulfide as the redox target of TrxA.

### 3.4. Light-Dark Modulation of FurA Thiol Oxidation 

**Figure 5:**
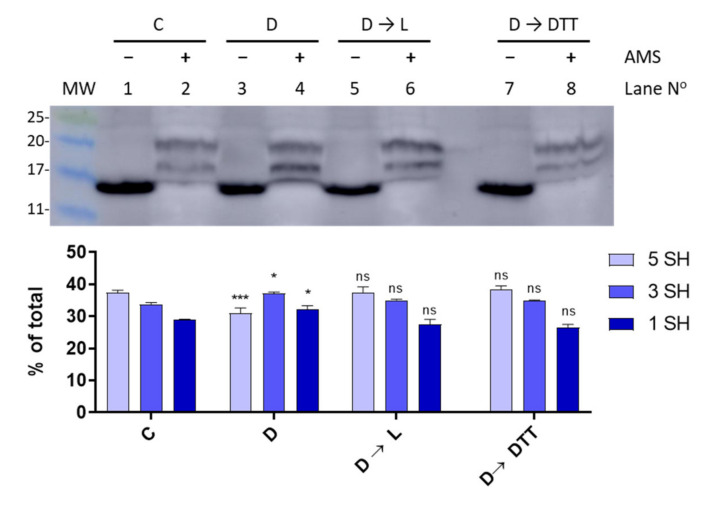
Light-dark modulation of FurA thiol oxidation. The in vivo redox status of FurA was assessed by Western blot under standard culture conditions of 30 μmol photons m^−2^ s^−1^ of white light (C), after 15 min of darkness (D), after 15 min of darkness followed by 3 h of exposure to 30 μmol photons m^−2^ s^−1^ of white light (D→L), and after 15 min of darkness followed by 3 h of exposure to 30 μmol photons m^−2^ s^−1^ of white light and 2 mM DTT (D→DTT). In all cases, *Anabaena* sp. PCC 7120 cultures were treated with 10% (*w*/*v*) trichloroacetic acid before being alkylated with 20 mM AMS. The relative abundance of each band was quantified by scanning densitometry and represented data are mean ± standard deviation of two biological replicates and two technical replicates. Significance was measured using one-way analysis of variance (ANOVA) comparing with the control (C): ^ns^ non significant, * *p* < 0.05, *** *p* < 0.00.

Since TrxA activity is connected to photosynthetically-reduced ferredoxin, we sought to investigate the thiol-redox dynamics of FurA under light to dark transitions. Thus, in order to assess the redox status of FurA in different light regimes, light-adapted cultures (30 μmol photons m^−2^ s^−1^) in the exponential phase of growth were placed in the dark for 15 min. Western blot analyses of *Anabaena* cells treated with AMS showed that the light to dark transition results in an evident oxidation of a fraction of FurA (Figure 5). As previously stated [20], under standard culture conditions in continuous light, FurA displays three different redox states. In these conditions, the predominant form of the regulator in the cell is the fully reduced form (upper band), as it can be stated after the treatment with AMS (Figure 5, lane 2). Conversely, after 15 min of darkness and treatment of *Anabaena* cells with AMS, the distribution of the FurA redox states changes and most of the FurA protein becomes partially oxidized, and around one third of the protein is completely oxidized, exhibiting two disulfide bonds (Figure 5, lane 4). Reillumination of the culture for 3 h leads to the recovery of the pool of reduced FurA (Figure 5, lane 6), which is consistent with the fraction of reduced protein observed after treatment with DTT of dark-adapted cultures (Figure 5, lane 8). 

## 4. Discussion

FurA is a multifunctional protein whose activity in cyanobacteria expands beyond iron-responsive transcriptional regulation. The absence of tightly coordinated, structural zinc in FurA from *Anabaena* sp. PCC7120, provides this regulator with additional properties compared to its orthologs in heterotrophic bacteria. Thus, FurA displays disulfide-reductase activity and possesses the features of a heme-sensing protein [18,20]. In vivo, FurA is found mainly as a monomer with its five cysteines in several redox states, suggesting that it undergoes redox regulation. Since thiol-disulfide exchange is an essential regulatory mechanism of cell metabolism that has special relevance in photosynthetic organisms, many efforts have been made to identify redox targets in plant chloroplast and cyanobacteria. Disulphide proteomics of *Synechocystis* sp. PCC6803 led to the identification of thioredoxin-linked processes involving both soluble and membrane proteins [37,38,39]. Furthermore, *Synechococcus* 7002 chemical profiling of temporal redox dynamics, in response to nutrient limitation, led to the identification of 176 proteins undergoing ~5−10-fold dynamic redox change, including FurA and Zur (FurB) [22]. These authors provided evidence that transient redox changes govern a broad range of biological processes, such as signal transduction, ROS remediation, photosynthesis, metabolism, and protein synthesis, among others. However, pretreatment of *Synechococcus 7002* with recombinant TrxA followed by in vitro probe labeling suggested that only one third of the proteins undergoing dynamic redox events involving cysteine residues were dependent on TrxA. Moreover, analysis of redox-sensitive proteins to light-dark transitions in *Synechococcus 7002* unveiled an even larger number of targets (~350 proteins) [23], though FurA was not identified in this study. A different proteomic approach to quantify and identify site-specific reversibly oxidized thiols in the cyanobacterium *Synechocystis* sp. PCC 6803 led to the conclusion that around one-third of the proteome underwent dynamic redox changes [21]. This study highlights the extent of redox modulation after light-to-dark transition, though the approach determines the level of total reversible thiol oxidation, including not only thiol-disulfide exchange but also potential S-glutathionylation, S-nitrosylation or S-sulfenylation. The occurrence of several redox-states of FurA in *Anabaena* cells is in good concordance with all these results [21,22,23]. However, none of them managed to identify TrxA as the ultimate reductant of FurA. Furthermore, FurA was also not found among the TrxA targets isolated using TrxA site-directed mutants preventing the resolution of mixed disulfides [37,38,39]. Conversely, the *E. coli* orthologue was identified as one of the *E. coli* thioredoxin targets [8,9], indicating that this regulation is not necessarily light-dependent. 

Our results provide evidence that FurA and TrxA interact in vivo, and the regulator is partially reduced by the latter. The fact that the reduction of FurA is not complete, neither in the dark nor in the in vitro system, is consistent with the data reported by Guo et al. [21], which shows how dynamic redox changes mostly correspond with/to partial activation or inactivation of specific proteins rather than work as all-or-nothing phenomena. Thus, mild redox modulation of FurA operated by TrxA adds an additional layer of complexity to the regulation of this master protein in *Anabaena* sp. PCC7120. Although the changes in the distribution of the three populations of FurA under different redox conditions in vivo are moderate, it should be considered that an important fraction of the FurA protein in the cell is bound to DNA and possibly less accessible to TrxA than the “free” regulator. We hypothesize that the interaction of FurA with DNA, together with the contact established between FurA monomers, could result in higher steric hindrance and diminishing TrxA accessibility, which would mainly modulate the redox state of the “free” unbound regulator. Signaling networks between thioredoxins and several cyanobacterial transcriptional regulators have been described [40,41,42,43] and the corresponding mechanism elucidated. The underlying purpose of the functional relationship between FurA and TrxA remains to be elucidated. The maintenance of a pool of fully reduced FurA in the cell could be essential either for the correct activity of the protein as a transcriptional regulator or for any of the other activities reported, including its capability to bind heme under the proper conditions. 

**Figure 6:**
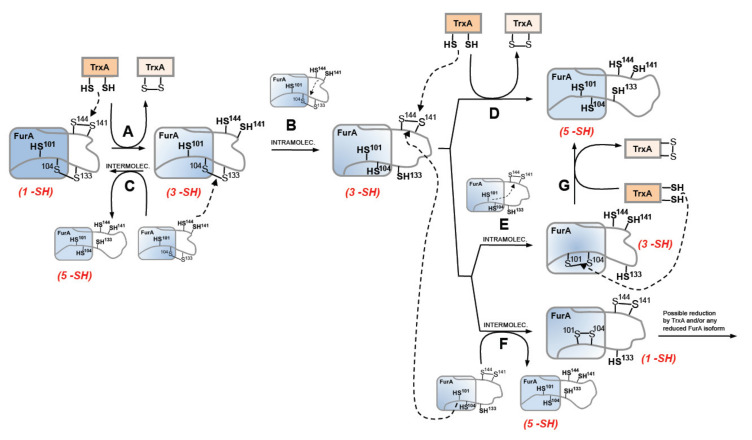
Proposed TrxA-mediated reduction mechanism of FurA from *Anabaena* sp. PCC7120. Arrowed dashed lines reflect the electron movement of the dithiol to the disulfide in each redox reaction, whereas arrowed solid lines denote the sequence in obtaining FurA isoforms in different oxidation states. Capital letters (A–G) identify the different redox processes involving FurA and TrxA that are explained in detail in the text.

As previously stated, our former knowledge about the architecture of the protein established that recombinant FurA monomers contain two intramolecular disulfide bridges, C104-C133 and C141-C144 [17]. The results presented herein suggest that the redox-active disulfide bond formed by C141 and C144 preferentially would be engaged in thiol disulfide exchange with reduced TrxA, since only the triple mutant C101-104-133S but not the C101-141-144S mutant is reduced by TrxA (Figure 6A). The second C104-C133 disulfide bond would serve to re-oxidize the reduced C141 and C144 pair of cysteines, either through an intra- or an intermolecular thiol-disulfide exchange reaction. Both possibilities (Figure 6B,C) appear feasible and are supported by the reductase activity displayed by the pair of cysteines C141 and C144 [20]. Moreover, according to a model of the three-dimensional structure of FurA [19], cysteines 104, 141, and 144 are found close to each other, which could be enough to allow intramolecular spontaneous disulfide interchange according to the proximity rule for thiol-disulfide exchange [44]. The occurrence of an intermolecular reaction is also supported by the finding of FurA as its own binding partner in pull-down assays using crude extracts from *Anabaena* in the presence of DTT, followed by matrix-assisted laser desorption/ionization-tandem mass spectroscopy (MALDI-MS/MS) analyses [20]. Reoxidation of the C141-C144 disulfide appears unavoidable because the disulfide C104-C133 in the triple mutant C101-C141-144S is not reduced by TrxA but, according to our alkylation results with AMS, reduction of FurA by TrxA leads to a significant fraction of the regulator with both disulfide bonds reduced. If reoxidation occurs via intramolecular transfer of the disulfide bond between C141-C144 and C104-C133, a new TrxA molecule would reduce the newly formed disulfide bond between C141 and C144 to obtain totally reduced FurA (Figure 6D). We do not rule out the possibility that the newly formed C141-C144 disulfide is also intra- (Figure 6E) or inter-molecularly reduced (Figure 6F), since we have previously reported that the reduced C101 and C104 pair also has reductase activity, unlike the C104 and C133 pair [20]. However, reduced FurA isoforms containing only three or one free –SH could be obtained respectively. We have verified that a triple mutant C103-C141-144S is also reduced by NTR-reduced TrxA, but less efficiently than the triple mutant C101-104-133S, according to alkylation results with AMS (Supplementary Material, Figure S1). This observation suggests that TrxA could also reduce the FurA isoform that contains C101-C104 disulfide bond, which would result in completely reduced FurA (Figure 6G). It cannot to be ruled out that the fully reduced protein may also contribute to the reduction of any of the partially reduced isoforms of FurA. Also, oxidized FurA isoform containing C101-C104 and C141-C144 disulfide bonds could be further reduced by TrxA and/or any FurA reduced isoform.

The reasons why TrxA preferentially would reduce C141-C144 disulfide but not C104-C133 disulfide can be varied involving the redox potential and pKa of the active CXXC motif in TrxA, the best molecular interaction, or also a conformational adjustment to orient the reactive groups to facilitate reaction among others [45]. In this sense, both the C141-C144 and C101-C104 disulfides in FurA have standard redox potentials of −238 mV and −235 mV, respectively, which fit with the redox potential of reducing TrxA that has a value of −271 mV, 33 mV and 36 mV more negative than that of each FurA disulfide pair, respectively [20,46]. Usually, lower redox potential (more negative) than the substrate is a condition for an oxidoreductase to donate electrons to or to cleave the disulfide in the substrate. Similar selective reduction by a Trx family member of one out of two disulfide bonds present in the same protein molecule has been reported for NADP-malate dehydrogenase (NADP-MDH), a chloroplast thiol-modulated enzyme from *Arabidopsis* [47]. NADP-MDH contains two redox-active cysteine pairs C77-C82 and C418-C430 in the N- and C- terminal regions, respectively. Whereas Trx-*f* can reduce both of the disulfide bonds, Trx-*m* can reduce either the N- or the C-terminal disulfide bridge alone [47]. According to our observations, the abundance of a particular population of reduced FurA isoform in the cyanobacterial cytoplasm would depend on the amount of reduced TrxA, which in turn would affect the redox status of different cysteines depending on the reduced FurA population. It should be taken into account that FurA cysteines are important not only for DNA-binding, but also for the interactions of this regulator with its co-repressor metal, as well as with heme and 2-OG [17,18,19]. Therefore, redox states of FurA cysteine residues appear to determine the ability of the regulator to sense different small molecules originated from metabolic activities and to integrate different types of information. Possibly these different redox forms of FurA still bind to DNA in vivo as long as they keep cysteine 101 in a reduced state. The subtle changes observed in the abundance of different FurA populations after light-dark modulation may reflect that not only Trx family members, but also small molecules associated to the different FurA isoforms could govern thiol switches in FurA and integrate its redox state and the metabolic state of the cell to respond appropriately to environmental challenges.

## 5. Conclusions

FurA is a multifunctional protein whose post-translational regulation seems rather complex. It has been proven that some of the roles played by this regulator rely on the redox status of its cysteines. Thus, the ability of FurA to bind Fe^2+^ and in turn to target DNAs depends on the thiol redox state of C101. Similarly, the effective interaction of FurA with 2-OG requires DTT. Furthermore, the disulfide-reductase activity displayed by both CXXC motifs present in the protein, together with the occurrence of several redox states of FurA in the cyanobacteria, raise the question of the identification of potential redox partners involved in the thiol-disulfide exchanges necessary to facilitate the interaction of FurA with its different ligands. Based on in vitro and in vivo evidences, TrxA emerges as a novel player in the maintenance of the different redox states exhibited by FurA. TrxA preferentially reduces the C141-C144 disulfide, while it is unable to reduce the C104-C133 pair. Reversible oscillation of FurA thiol-disulfide states in response to light/dark transitions links either directly or indirectly the different activities of the regulator to the activity of the photosynthetic electron transport chain. Nevertheless, it cannot be ruled out that some other partner, including any of the nine thioredoxin ORFs identified in the *Anabaena* sp. PCC7120 genome, could also participate in the redox modulation of FurA. 

## Data Availability

Data is contained within the article.

## Supplementary Materials

The following are available online at https://www.mdpi.com/article/10.3390/antiox10060913/s1, Thioredoxin Dependent Changes in the Redox States of FurA from *Anabaena* sp. PCC 7120/s1, Figure S1: Controls of the cross-linking reaction between FurA and TrxA, Figure S2: Reconstitution of the pathway of electron transfer NADPH->NTR->TrxA->FurA shows that FurA is reduced by TrxA, Figure S3: Reduction of FurA C101-C104 disulfide bridge by TrxA.
