# Supplementary figures and images for: Thioredoxin Dependent Changes in the Redox States of FurA from Anabaena sp. PCC 7120
Source: Antioxidants (Basel). 2021 Jun 4;10(6):913. doi: 10.3390/antiox10060913 (PMC8229018; doi:10.3390/antiox10060913)

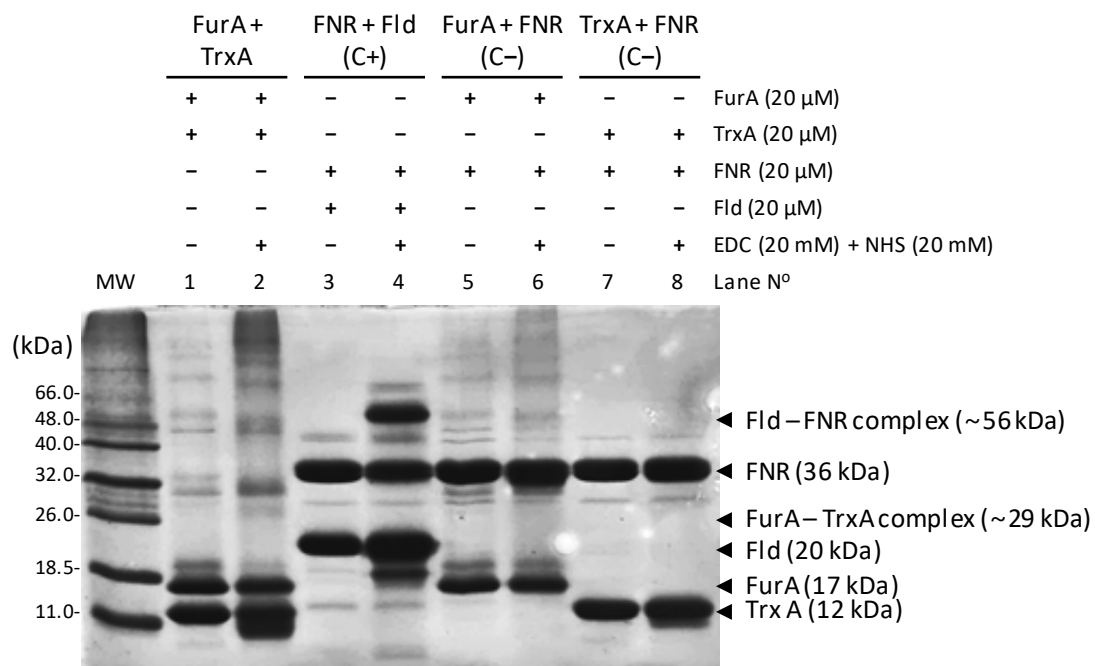

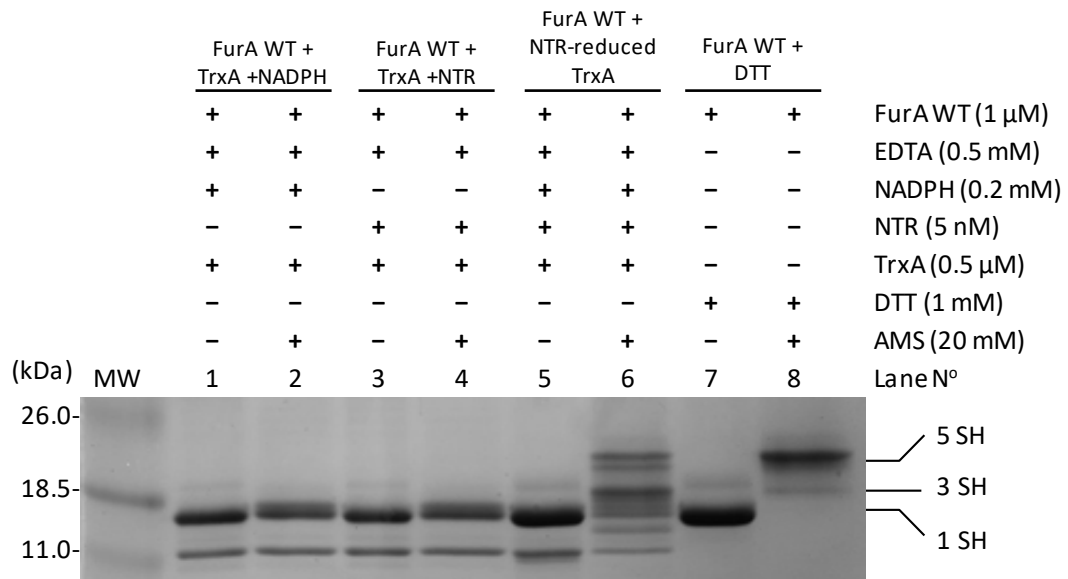

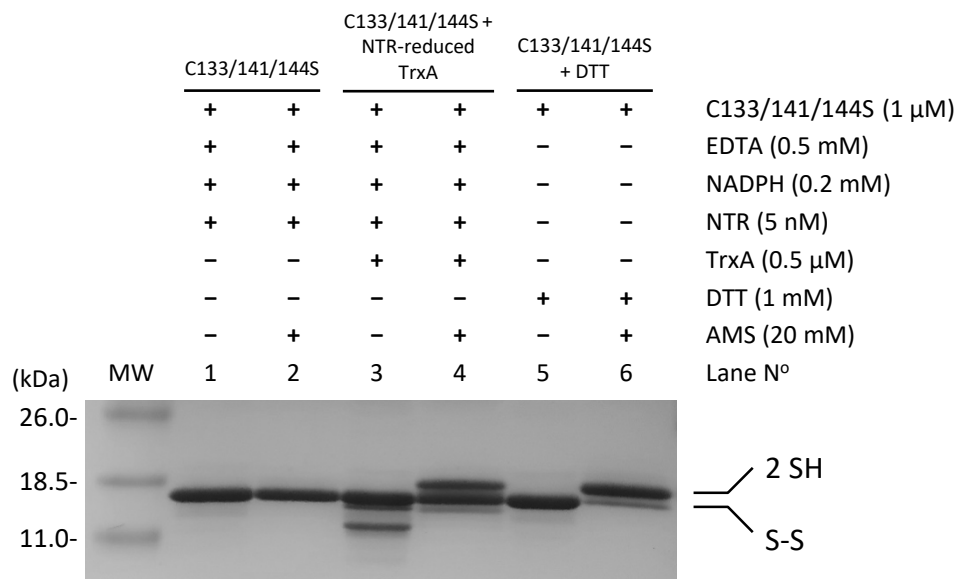

Supplement: Supplementary file 1 [file antioxidants-10-00913-s001.zip › antioxidants-1229206-supplementary.pdf]
